# Using the consolidated framework for implementation research to identify church leaders’ perspectives on contextual determinants of community-based colorectal cancer screening for Black Kentuckians

**DOI:** 10.1186/s43058-024-00621-9

**Published:** 2024-07-25

**Authors:** Aaron J. Kruse-Diehr, Derek Cegelka, Carlee Combs, Rose Wood, Elizabeth Holtsclaw, Jerod L. Stapleton, Lovoria B. Williams

**Affiliations:** 1 University of Kentucky College of Medicine, Lexington, KY USA; 2 University of Kentucky Markey Cancer Center, Lexington, KY USA; 3 Center for Implementation, Dissemination & Evidence-based Research, University of Kentucky, Lexington, KY USA; 4 Hawai’i Pacific University College of Nursing, Honolulu, Hawai’i USA; 5 University of Kentucky College of Public Health, Lexington, KY USA; 6 American Cancer Society, Lexington, KY USA; 7 University of Kentucky College of Nursing, Lexington, KY USA

**Keywords:** African Americans, Church-based health promotion, Colorectal cancer, Consolidated framework for implementation research, Screening, Stool-based screening

## Abstract

**Background:**

Black Kentuckians experience more deleterious colorectal cancer (CRC) outcomes than their White counterparts, a disparity that could be reduced by increased screening in Black communities. Previous research has shown that Black Kentuckians may not be equitably informed of different CRC screening options by health care providers, making community-based screening a potentially effective option among this disparate population. We used the Consolidated Framework for Implementation Research (CFIR) to identify church leaders’ perspectives of contextual factors that might influence community-based screening and explore the feasibility of using church-based screening outreach.

**Methods:**

Six participants were selected, based on leadership roles and interest in CRC screening, from five established Louisville-area church partners that had previously participated in community health initiatives. Data were collected, both virtually and in-person, in Summer 2021 using semi-structured interview guides developed with guidance from the CFIR Guide that focused on domains most relevant to community-based interventions. Data were transcribed verbatim, coded by two independent researchers, and member checked for accuracy.

**Results:**

Data were aligned primarily with six CFIR constructs: key stakeholders, champions, opinion leaders, tension for change, compatibility, and culture. Participants noted a strong tension for change in their community due to perceptions of inadequacy with clinical approaches to CRC screening. Additionally, they stressed the importance of identifying individuals both within the church who could champion CRC screening and help implement program activities, as well as those outside the church who could collaborate with other local organizations to increase participant reach. Finally, participants agreed that faith-based CRC screening aligned with church culture and would also likely be compatible with overall community values.

**Conclusions:**

Overall, our church partners strongly endorsed the need for, and importance of, community-based CRC screening. Given a history of successful implementation of health promotion programs within our partner churches, it is highly likely that a CRC screening intervention would also be effective. Findings from this study will be used to identify implementation strategies that might positively impact a future faith-based CRC screening intervention, as well as CFIR constructs that are most positively associated with CRC screening completion.

Contributions to the literature

- Traditional health care settings may not offer Black patients equitable colorectal cancer screening options, making the provision of screening opportunities in nontraditional settings particularly important in Black communities.
- We found that leaders of Black churches in Kentucky are highly receptive to faith-based screening education and distribution of at-home stool-based tests to promote increased colorectal cancer screening in their communities.
- To increase the likelihood of successful implementation and ensure adequate intervention reach, church partners suggested identifying individuals both within and outside of the faith community.

## Introduction

Black Kentuckians have higher colorectal cancer (CRC) incidence and mortality rates than White residents [1], and screening remains one of the most modifiable ways to reduce screening disparities. Research has shown that high screening utilization can eliminate Black-white screening disparities, equalize incidence rates, increase the percentage diagnosed with local (vs. advanced or regional) stage CRC, and substantially reduce the racial mortality gap [2]. Despite an increase in CRC screenings among Black Kentuckians during the past decade, the Black-white CRC incidence and mortality disparity in Louisville (46.5 vs. 42.1 per 100,000 population and 20 vs. 12.3 per 100,000 population, respectively) remains substantially higher than the US Black vs. white rates [1]. About half of Kentucky’s Black population resides in Louisville [3], where despite the existence of multiple resources to support early cancer detection and treatment, over a quarter of Black Louisville residents, aged 50–75, remained unscreened as of 2018 [4].

The US Preventive Services Task Force recommends CRC screening beginning at age 45, with choice of test dependent on risk factors. Stool-based CRC screening, such as the fecal immunochemical test (FIT), is recommended [5] for individuals at average risk for CRC because it is inexpensive [6], noninvasive, and convenient given that it can be completed in one’s own home. Furthermore, it reduces several individual-level and structural barriers to screening, such as stigma related both to perceived test invasiveness and masculinity (among males), transportation issues, and required time off work [7, 8]. Nevertheless, stool-based screening rates are lower in Black than in white populations [9], despite clinical trials showing Black patients are more likely to get screened when offered FIT compared to colonoscopy [10, 11]. Previous research has suggested that despite recognizing the importance of regular CRC screening, Black Kentuckians may not be aware that there are screening options beyond colonoscopy [12]. Data from the Health Information National Trends Survey (HINTS), a weighted nationally representative mailed survey on cancer-related health communication trends, has similarly found that health care providers do not offer CRC screening options to Black patients at the same rate as white individuals [13]. Although providers and health care delivery systems have clear roles in recommending and providing screening, multiple barriers keep Black individuals from being screened, including social determinants of health, medical distrust, perceived test invasiveness, fear of pain, and fatalism [14–17]. It is also possible that implicit bias prevents health care providers from regularly informing their average-risk Black patients of different screening options [12], making community-based screening outreach a health equity priority.

Church-based health promotion (CBHP) is effective in Black communities [18], and faith-based interventions have proven successful at increasing CRC screenings among Black individuals [19–21]. Nevertheless, there is a need to identify contextual determinants of CRC screening when considering implementation in a new community or population. To better understand the context for planning and implementing a community-based CRC screening outreach program, our research team—comprised of investigators from [blinded comprehensive cancer center] and [blinded regional organization]—conducted interviews with six key informants from Louisville-area Black churches to identify their beliefs about multifactorial facilitators and barriers to CRC screening among Black Kentuckians. Guided by the Consolidated Framework for Implementation Research ( [22]; CFIR), we explored key informants’ perceptions of CRC screening; their confidence in their organization/faith community’s ability to implement a FIT distribution intervention; their own knowledge, beliefs, and self-efficacy to complete CRC screening; external influences that might affect intervention success; and necessary roles for themselves and others throughout the intervention.

## Methods

### Setting

This study was conducted in collaboration with five mostly small Louisville-area churches initially chosen because of historical involvement in community health initiatives (e.g., mobile mammography, blood pressure, diabetes screening) and willingness to participate in research. Key informants from each church were identified and purposively selected based on (a) church leadership roles (e.g., in charge of health ministry or community outreach activities), (b) specific interest in CRC (screening), and/or (c) ability to participate. Because they held leadership roles within the church, key informants were uniquely positioned to provide detailed information about church structure, common beliefs and values, and other important factors that could either impede or facilitate the implementation of a church-based CRC screening program.

### Data collection

Data were collected in Summer 2021 via six one-on-one interviews (conducted both in-person and via Zoom) that lasted between 30–45 min. Semi-structured interview guides (22 questions, plus probes) were developed using the CFIR Guide [23] and focused on domains that were (a) most relevant to community-based (rather than clinical) interventions (e.g., *intervention characteristics, inner setting, characteristics of individuals, outer setting,* and *process*) and (b) would be applicable/answerable by participants given their church. For example, questions included those about identification of project stakeholders, opinion leaders, and implementation leaders; overall culture of the church and intervention compatibility; and community and church needs related to CRC screening and CRC in general. Upon completion, project participants were provided a $20 gift card for their participation.

### Data analysis

Interviews were facilitated by the project’s principal investigator and audio recorded before being sent to a professional transcriptionist. Approximately 10 random snippets of audio were compared to the transcripts to ensure accuracy. Two members of the research team (RW, CC) trained in qualitative research individually coded transcripts to a priori defined CFIR domains based on a template made publicly available by the CFIR authors [23] and met weekly to ensure consistency of coding/categorization. In the few occasions where conflicts arose, the study’s principal investigator (AK-D) mediated to build consensus. To minimize interpretation bias, a subset of project participants was presented via email with a table of thematic coding summaries to ensure we accurately interpreted participant data, and no major changes were suggested throughout this process.

## Results

### Description of participants

Participants all held leadership roles in their respective church and included members of the ministerial team, health ministry, and church elders. All identified as Black or African American, had at least some postsecondary education, were insured by either an employer plan or Medicare, and ranged in age from 41 to 72 years old. Table 1 displays CFIR domains and relevant quotes for each domain.

**Table 1:** CFIR domains and representative quotes from church leaders

Tension for Change	I think it's a strong need in the community simply because many people do not take their health as a priority, or some people don't like doctors
	Absolutely there is [a need for community-level screening]. So that more people can be educated about the screening process and early detection…[a]nd I think they'll be receptive to it
Compatibility	[The program would be compatible] because we've had different health types of programs previously that people participated in
	This is only my personal belief is that, if our church would be the one that is having it and promoting it, I believe that if they have a compassion to do it, then selling it to the community or promoting it in the community, it would be a positive thing
Culture	If it's OK for [the pastor] to let it happen at his church, it's got to be OK, because he's not going to let everything come up in there
	Well, we've been doing the mobile mammogram for years. We have a couple of nurses at the church and nurse practitioners that whenever there is [an awareness] month. Last month was domestic violence and breast cancer, so we speak about that. Our pastor gives us a platform and he allow us to speak on certain topics, speak on certain things
Opinion leaders	Testimonials, somebody that has lived through this experience, some may have overcome and some may not. So, family members that can say, "yes, please get this done. My such and such, they didn't make it. And I wished they would have, because they waited too late to get screened."
	The Nurses Guild which includes our nurses, our nurse practitioners. We have a few. The associate ministers. Reverend [name redacted], he's one of the associate ministers. He's a survivor. Deacons, because we've got three of the gentlemen that were there are survivors. And just the survivors in the church
Champions	It would have to be, honestly, somebody that looks like us. And they would have some experience either, with the medical field and you would have to respect their time
	In this community, women do drive a lot of decision making. And having a woman as a champion, because that was one of the things I was going to come to, having a woman as a champion of this program will get a better participation than having a guy as a champion of it, I think
Key Stakeholders	I don't have a huge social circle, but a friend of mine…does a college fair directed to Blacks and minorities… Somebody like her would be perfect [as a key stakeholder]
	You talked about the fraternities and sororities and the NAACP, both of those are great platforms
	I would also reach out to the Barber's Union. There's several quasi groups, as you know, I mean, getting them, especially a women's side of it all

### Key stakeholders

Participants defined the construct of key stakeholders as both a designation for people and groups with influence over community opinion that regularly interacted with the local Black community in some way, both within and apart from church membership. Key stakeholders used multiple communication forms to ensure all age groups receive messaging about community health programs, including texting, flyers, radio ads, written and spoken church announcements, general word-of-mouth, newspapers, and emails. Examples of groups included barber’s unions, NAACP members, and Black fraternities and sororities. Organizations not associated with churches were identified as key stakeholders due to their established communication channels for outreach to community members who may not participate in church activities.

### Champions

Unlike key stakeholders, who were identified as local sources of influence, participants described champions as members of the community who could help with project implementation in a notable way. These individuals were defined as champions because of their dedication to the community outside of formal duties and history. Some commonly described characteristics included personal interest in the research topic, history of community engagement, and leadership and communication skills. Often, they had previously organized or assisted with community outreach programs, such as college fairs for minority students, mobile breast cancer screenings, clothing drives, and church programs. Interviewees noted that trusted champions are key to community engagement for any future cancer-related programming and that these types of programs need to be marketed broadly within the community in conjunction with identified key stakeholders.

### Opinion leaders

Like key stakeholders, opinion leaders also held influence over the opinion of the community; however, their influence was related to having a specific skillset or level of knowledge related to a particular health topic. Participants noted that opinion leaders were not necessarily formally appointed or famous, but that their medical training or experience with a health topic is generally valued by members of the community. To that end, doctors, nurses, and cancer survivors from the community were all often listed as examples, as were members of the community who had lost a family member to CRC because the emotional pull of their personal testimonies might be useful in motivating screening behavior. Opinion leaders were described as serving in both formal (i.e., church leadership, community organizations) and intrapersonal (i.e., one-on-one) settings.

### Tension for change

Tension for change is typically described as the degree to which stakeholders perceive a current situation as intolerable or needing change. Participants described their community’s tension for change as originating from both medical disparities and community needs and suggested community members might be more receptive to receiving cancer screening information in trusted locations such as community agencies/groups or churches rather than in traditional health care settings. Furthermore, interviewees frequently noted that members of their community were rarely given screening options beyond colonoscopy by health care providers. Given these identified inequities in traditional health care settings, interviewees strongly endorsed community-level screening.

### Compatibility

Compatibility is typically defined as the degree of fit between meaning and values attached to the intervention by those involved, as well as how the intervention aligns with individuals’ values and needs and existing system workflows. Given that other church-sponsored outreach programs were historically well-received by the community, participants believed community-based CRC screening would be received similarly. Relatedly, participants frequently referred to a sense of duty in terms of promoting health and wellbeing of both their fellow church parishioners as well as the community at large. While participants underscored the importance of individual responsibility for one’s health, they also highlighted the need for population health and appropriate channels for delivering important health communication.

### Culture

Finally, the construct of culture broadly relates to the norms, values, and basic assumptions of a given organization [22]. Participants described their church culture as one in which health promotion programs are typically approved by church leadership before being implemented to ensure that any health outreach program aligns with the values and beliefs of the church and its members. Because community members recognize this process, the established culture of the church lends ethos to programs or interventions that the church chooses to implement or endorse. Participants also referenced the larger culture of the Black community and how it might facilitate successful implementation of a community-based CRC screening program, noting the history of medical injustice/inequity and how it has affected the community. As a result, interviewees discussed the importance of community mobilization with respect to CRC and screening importance.

## Discussion

We used the CFIR to understand the context for planning and implementing a church-based CRC screening outreach program. CBHP allows for a collaborative approach in reducing health disparities and has been effective on multiple health behaviors within the Black community [18]. Because churches have historically served their communities, they are positioned to be prime settings for public health programming. For CRC, in particular, research has shown that spiritually based or church-led interventions increase CRC screenings among Black individuals [19–21]. Churches can be instrumental in participant recruitment for health interventions because of their resources, access to specific populations, and frequent inclusion of health as part of their missions or respective ministries [18]. The CFIR is frequently used in clinical settings to explain why implementation may succeed or fail [22]; however, its application is also particularly useful for planning community-based interventions, especially if clinical settings do not provide equitable opportunities for CRC screening [12], as indicated in our findings.

Participants voiced that community members would likely be more receptive to cancer screenings in trusted community locations; nevertheless, faith-based partners must value the importance of CRC screenings to ensure intervention success [24, 25], buy-in that is likelier to occur when the health issue aligns with the church’s overall culture and there is a strong tension for change. Participants in this study routinely expressed concern that their screening needs were not being adequately addressed in clinical settings. Additionally, church leadership, including deacons and members of the ministerial team, were themselves CRC survivors, lending “top-down” intervention support, a finding aligned with previous studies of faith-based organizations that featured supportive leaders and overall culture [26–28]. Furthermore, CRC screening was identified as being compatible with community members’ values based on the successes of previous faith-based health promotion activities. This finding is critical, given that a track record of successful church-based health promotion often yields greater success for future programs [18, 29], along with the formation of partnerships with other faith organizations [30]. Ultimately, to achieve optimal outcomes, it is critical that researchers identify churches with “cultures of concern” whose inner settings reflect the importance of cancer screening.

The determinants identified from this study can be used to identify implementation strategies that leverage church and community strengths to implement a community-based CRC program. For example, previous research has recommended providing health behavior change training and capacity building to support adoption and implementation for pastors and staff [31–33]. These sorts of strategies might be most useful in the early tailoring and adaptation processes of community-based CRC screening interventions. While health promotion activities are not necessarily unique in faith-based settings, churches may be more familiar with educational programs or physical activity/diet interventions rather than cancer screening [18, 34, 35]. Although our plan for future intervention research includes churches partnering with local organizations trained in conducting CRC screening activities, church partners will still need to take an active role in implementation. Through this interventional work, we will score CFIR constructs to identify constructs most associated with positive and negative, as well as weak or strong, influences on implementation. In a weight management study, for example, tension for change was one of the ten CFIR constructs strongly associated with greater implementation success, while positive trends were also found for champions and implementation leaders [36]. Identifying constructs with strong positive influences on CRC screening is critical to inform future scale-up of community-based screening interventions.

### Limitations

This study’s findings should be interpreted with consideration of its limitations. First, our sample size was small, and participants were derived from a pool of the study team’s previous collaborators, meaning our findings may not be generalizable to other Black churches or faith communities, even those in Louisville. Additionally, given the history of collaboration, it is possible that participants provided more socially desirable responses, though we attempted to mitigate this risk of bias via member checking and multiple investigator debriefings. Second, it is possible that the beliefs of church leadership might not align with the needs or beliefs of community members who would receive screening services or that some community members might not be well-connected with the church; in this case, it is critical that the church leverage other community partners, as they described in identifying key stakeholders. Similarly, while participants noted value and cultural alignment for the implementation of a future community-based CRC intervention, our church partners varied in terms of available resources, which could likely lead to differences in overall clinical and implementation outcomes. In these cases, it might be worthwhile to explore partnerships in which churches could simultaneously leverage each other’s strengths and potentially reach a larger population with screening activities, including partnering with churches and organizations that are newer to implementing outreach programs. Finally, except for one participant, our sample skewed older (60 years of age and older), and findings may not be representative of all age groups. Since the USPSTF-recommended CRC screening age has been reduced from 50 to 45 years old for individuals at average risk, it is important to ensure that values endorsed by older churchgoing adults are congruent with younger individuals eligible for screening.

## Conclusion

The establishment of partnerships with Black churches to promote CRC screening education and FIT distribution may represent a promising approach to community-based CRC screening, particularly in locations where Black Kentuckians broadly perceive disparities in clinical screening opportunities. Leveraging the history of the Black church as a trusted center for community support and empowerment is critical to promote sustainment of CRC screening activities and reducing disparities.

## Data Availability

The data used in this research (i.e., transcripts from in-depth interviews) are not publicly available due to the small sample and concerns about confidentiality.

## Abbreviations

CBHP: Church-based health promotion
CFIR: Consolidated Framework for Implementation Research
CRC: Colorectal cancer
FIT: Fecal immunochemical test
NAACP: National Association for the Advancement of Colored People
USPSTF: United States Preventive Services Task Force

## References

[1] Kentucky Cancer Registry. Based on data released; 2023. Available from: https://www.cancer-rates.info/ky/.

[2] Grubbs SS, Polite BN, Carney J Jr, Bowser W, Rogers J, Katurakes N, et al. Eliminating racial disparities in colorectal cancer in the real world: it took a village. J Clin Oncol. 2013;31(16):1928–30. 23589553 10.1200/JCO.2012.47.8412 PMC3661932

[3] United States Census Bureau. QuickFacts: Jefferson County, Kentucky. Available from: https://www.census.gov/quickfacts/fact/table/jeffersoncountykentucky,KY/. Accessed 14 Feb 2024.

[4] Kentucky Department for Public Health (KDPH) and Centers for Disease Control and Prevention (CDC). Kentucky Behavioral Risk Factor Survey Data. Cabinet for Health and Family Services, Kentucky Department for Public Health; 2018.

[5] US Preventive Services Task Force, Davidson KW, Barry MJ, et al. Screening for colorectal cancer: US Preventive Services Task Force recommendation statement. JAMA. 2021;325(19):1965–77. 34003218 10.1001/jama.2021.6238

[6] Subramanian S, Tangka FK, Hoover S, Cole-Beebe M, Joseph D, DeGroff A. Comparison of program resources required for colonoscopy and fecal screening: findings from 5 years of the colorectal cancer control program. Prev Chronic Dis. 2019;16:180338. 10.5888/pcd16.180338 PMC6513474 31022371

[7] Adams LB, Richmond J, Corbie-Smith G, Powell W. Medical mistrust and colorectal cancer screening among African Americans. J Community Health. 2017;42(5):1044–61. 28439739 10.1007/s10900-017-0339-2 PMC5654700

[8] Brooks E, Islam JY, Perdue DG, et al. The Black Panther, masculinity barriers to medical care, and colorectal cancer screening intention among unscreened American Indian/Alaska Native, Black, and white men. Front Public Health. 2022;10:814596. 35462819 10.3389/fpubh.2022.814596 PMC9019156

[9] Shapiro JA, Klabunde CN, Thompson TD, Nadel MR, Seeff LC, White A. Patterns of colorectal cancer test use, including CT colonography, in the 2010 National Health Interview survey. Cancer Epidemiol Biomarkers Prev. 2012;21(6):895–904. 22490320 10.1158/1055-9965.EPI-12-0192 PMC4489134

[10] Inadomi JM, Vijan S, Janz NK, Fagerlin A, Thomas JP, Lin YV, et al. Adherence to colorectal cancer screening: a randomized clinical trial of competing strategies. Arch Intern Med. 2012;172(7):575–82. 22493463 10.1001/archinternmed.2012.332 PMC3360917

[11] Gupta S, Halm EA, Rockey DC, Hammons M, Koch M, Carter E, et al. Comparative effectiveness of fecal immunochemical test outreach, colonoscopy outreach, and usual care for boosting colorectal cancer screening among the underserved: a randomized clinical trial. JAMA Intern Med. 2013;173(18):1725–32. 23921906 10.1001/jamainternmed.2013.9294 PMC5228201

[12] Kruse-Diehr AJ, Cegelka D, Holtsclaw E, Stapleton J, Burnett C, Wood R, et al. Barriers and facilitators to stool-based screening for colorectal cancer among Black Louisville residents. J Cancer Educ. 2023;38(3):1050–8. 36301412 10.1007/s13187-022-02231-2 PMC10501315

[13] Lee TC, Mathis AL, Dutton MT. An examination of early colorectal cancer screening guidelines for African Americans: hints from the HINTS data. J Health Disparities Research and Practice. 2016;9(1):175–81.

[14] Greiner KA, Born W, Nollen N, Ahluwalia JS. Knowledge and perceptions of colorectal cancer screening among urban African Americans. J Gen Intern Med. 2005;20(11):977–83. 16307620 10.1007/s11606-005-0244-8 PMC1490251

[15] Williams R, White P, Nieto J, Vieira D, Francois F, Hamilton F. Colorectal cancer in African Americans: an update prepared by the Committee on Minority Affairs and Cultural Diversity, American College of Gastroenterology. Clin Transl Gastroenterol. 2016;7(7): e185. 27467183 10.1038/ctg.2016.36 PMC4977418

[16] May FP, Whitman CB, Varlyguina K, Bronley EG, Spiegel BMR. Addressing low colorectal cancer screening in African Americans: using focus groups to inform the development of effective interventions. J Cancer Educ. 2016;31(3):561–74. 10.1007/s13187-015-0842-z PMC4644112 25963898

[17] Sly JR, Edwards T, Shelton RC, Jandorf L. Identifying barriers to colonoscopy screening for nonadherent African American participants in a patient navigation intervention. Health Educ Behav. 2013;40(4):449–57. 23086556 10.1177/1090198112459514 PMC3695062

[18] Campbell MK, Hudson MA, Resnicow K, Blakeney N, Paxton A, Baskin M. Church-based health promotion interventions: evidence and lessons learned. Annu Rev Public Health. 2007;28:213–34. 17155879 10.1146/annurev.publhealth.28.021406.144016

[19] Holt CL, Litaker MS, Scarinci IC, Debnam KJ, McDavid C, McNeal SF, et al. Spiritually based intervention to increase colorectal cancer screening among African Americans: screening and theory-based outcomes from a randomized trial. Health Educ Behav. 2013;40(4):458–68. 23033548 10.1177/1090198112459651 PMC5568036

[20] Leone LA, Allicock M, Pignone MP, Walsh JF, Johnson L-S, Armstrong-Brown J, et al. Cluster randomized trial of a church-based peer counselor and tailored newsletter intervention to promote colorectal cancer screening and physical activity among older African Americans. Health Educ Behav. 2016;43(5):568–76. 26515276 10.1177/1090198115611877 PMC5963515

[21] Jandorf L, Braschi C, Ernstoff E, Wong CR, Thelemaque L, Winkel G, et al. Culturally targeted patient navigation for increasing African Americans’ adherence to screening colonoscopy: a randomized clinical trial. Cancer Epidemiol Biomarkers Prev. 2013;22(9):1577–87. 23753039 10.1158/1055-9965.EPI-12-1275 PMC3769457

[22] Damschroder LJ, Aron DC, Keith RE, Kirsh SR, Alexander JA, Lowery JC. Fostering implementation of health services research findings into practice: a consolidated framework for advancing implementation science. Implement Sci. 2009;4:50. 19664226 10.1186/1748-5908-4-50 PMC2736161

[23] Consolidated Framework for Implementation Research (CFIR) Guide; 2019. https://cfirguide.org. 10.1002/lrh2.10402 PMC11019373 38633023

[24] Teal R, Moore AA, Long DG, Vines AI, Leeman J. A community-academic partnership to plan and implement an evidence-based lay health advisor program for promoting breast cancer screening. J Health Care Poor Underserved. 2012;23(2 Suppl):109–20. 22643559 10.1353/hpu.2012.0076 PMC12278316

[25] Breslau ES, Weiss ES, Williams A, Burness A, Kepka D. The implementation road: engaging community partnerships in evidence-based cancer control interventions. Health Promot Pract. 2015;16(1):46–54. 24700166 10.1177/1524839914528705

[26] Castañeda SF, Holscher J, Mumman MK, Salgado H, Keir KB, Foster-Fishman PG, et al. Dimensions of community and organizational readiness for change. Prog Community Health Partnersh. 2012;6(2):219–26. 22820232 10.1353/cpr.2012.0016 PMC4169887

[27] Maxwell AE, Santifer R, Chang LC, Gatson J, Crespi CM, Lucas WA. Organizational readiness for wellness promotion—a survey of 100 African American church leaders in South Los Angeles. BMC Public Health. 2019;19(1):1–10. 31101096 10.1186/s12889-019-6895-x PMC6525409

[28] Tagai EK, Scheirer MA, Santos SLZ, Haider M, Bowie J, Slade J, et al. Assessing capacity of faith-based organizations for health promotion activities. Health Promot Pract. 2018;19(5):714–23. 29058956 10.1177/1524839917737510 PMC5878962

[29] Kramish Campbell M, James A, Hudson MA, Carr C, Jackson E, Oakes V, et al. Improving multiple behaviors for colorectal cancer prevention among African American church members. Health Psychol. 2004;23(5):492. 15367069 10.1037/0278-6133.23.5.492

[30] McNeill LH, Reitzel LR, Escoto KH, Roberson CL, Nguyen N, Vidrine JI, et al. Engaging Black churches to address cancer health disparities: project CHURCH. Front Public Health. 2018;6:191. 30073158 10.3389/fpubh.2018.00191 PMC6060541

[31] Bernhart JA, Dunn CG, Wilcox S, Saunders RP, Sharpe PA, Stucker J. Church leaders’ barriers and facilitators before and after implementing a physical activity and nutrition intervention. Health Educ Res. 2019;34(2):188–99. 30601982 10.1093/her/cyy051

[32] Haughton J, Takemoto ML, Schneider J, Hooker SP, Rabin B, Brownson RC, et al. Identifying barriers, facilitators, and implementation strategies for a faith-based physical activity program. Implement Sci Commun. 2020;1:51. 32885207 10.1186/s43058-020-00043-3 PMC7427873

[33] Leyva B, Allen JD, Ospino H, Tom LS, Negrón R, Buesa R, et al. Enhancing capacity among faith-based organizations to implement evidence-based cancer control programs: a community-engaged approach. Transl Behav Med. 2017;7(3):517–28. 28733726 10.1007/s13142-017-0513-1 PMC5645291

[34] Wilcox S, Jake-Schoffman DE, Saunders RP, Kinnard D, Kaczynski AT, Hutto B, et al. Predictors of implementation in the Faith, Activity, and Nutrition dissemination and implementation study: application of the Consolidated Framework for Implementation Research (CFIR) in a statewide initiative. Transl Behav Med. 2021;11(2):419–29. 32221601 10.1093/tbm/ibaa025 PMC8496554

[35] Whitaker DE, Snyder FR, San Miguel-Majors SL, Bailey LO, Springfield SA. Screen to Save: Results from NCI’s Colorectal Cancer Outreach and Screening Initiative to Promote Awareness and Knowledge of Colorectal Cancer in Racial/Ethnic and Rural Populations [published correction appears in Cancer Epidemiol Biomarkers Prev. 2022 Jan;31(1):298]. Cancer Epidemiol Biomarkers Prev. 2020;29(5):910–7. 32358038 10.1158/1055-9965.EPI-19-0972 PMC12306618

[36] Damschroder LJ, Lowery JC. Evaluation of a large-scale weight management program using the consolidated framework for implementation research (CFIR). Implement Sci. 2013;8:51. 23663819 10.1186/1748-5908-8-51 PMC3656778

